# Identifying Needs and Support Services for Family Caregivers of Older Community-Based Family Members: Mixed-Method Research Findings

**DOI:** 10.1177/07334648241308726

**Published:** 2025-01-09

**Authors:** Donna M. Wilson, Jennifer Heron, Gilbert Banamwana

**Affiliations:** 1 3158 University of Alberta, Edmonton, AB Canada

**Keywords:** family caregiver/carer, community-based, home care, Canada, burnout, policy

## Abstract

A recent Canadian study conducted in one province identified family caregiver support needs and essential support services when caring for older community-based family members requiring assistance with activities of daily living. Weekly interviews of 150 volunteer caregivers over 6 months identified 11 support needs and 5 essential support services. Scoping literature reviews of the 11 needs found they had all been identified before. Program logic investigations of the 5 support services identified a patch-work of temporarily available support services in existence across the province. Two governmental policies are recommended: (a) provincial policy assuring access to the five support services, and (b) federal policy for federal-provincial funding transfers to address the provincial cost of assured community-based support services. Family caregivers require this support to maintain their own and their family member’s well-being, particularly as this caregiving prevents or delays older family member hospitalizations and nursing home entry.

**What this paper adds to the existing literature**

• Highlights the largely unacknowledged roles and contributions of family caregivers.
• Eleven family caregiver support needs were identified, and these are distinct from care recipients.
• Five essential support services were identified for policy and service action.

**Application to gerontological practice, policy, and/or research**

• Family carers of older people are greatly impacted by their caregiving.
• The work of family caregivers is essential for preventing or delaying hospital and nursing home admissions.
• Community-based supports for family caregivers should be assured.

## Introduction

The population aging that is occurring worldwide is a major historical milestone, as most people now are living to old age (65+ years) and therefore have the option of achieving important personal, family, and societal roles (Charles et al., 2017; Mather et al., 2015; Statistics Canada, 2018; Sunzi et al., 2023; Teixeira et al., 2020). Advanced aging is also occurring, with many more people reaching 85+ years of age (Alberta Department of Health, 2021; Charles et al., 2017; Statistics Canada, 2022). This is a critical age, as the upper age span of 85+ is marked by increased biological frailty and an escalating risk of both morbidity and mortality (Ringer et al., 2017; Schulz, 2013; Schulz et al., 2020; Serrano et al., 2017). It is common to refer to this group as the frail-elderly as they typically need help with activities of daily living, activities previously accomplished independently (Adams et al., 2013; Ringer et al., 2017; Schmidle et al., 2022; Statistics Canada, 2022). Although all developed countries have options for housing disabled adults, such as nursing homes and assisted living facilities, most older people avoid institutional living through the help of one or more family caregivers (Campbell-Enns et al., 2023; Compton et al., 2020; Herbert & Molinsky, 2019; Lilly et al., 2012; Siegler et al., 2015).

For decades, the supportive work of family caregivers has been recognized as important (Campbell-Enns et al., 2023; Gitlin & Wolff, 2011; Schulz et al., 2020; Sherman, 2019). It is also well known as physically and emotionally exhausting (Gibbons et al., 2014; Zaalberg et al., 2023). Burnout and other negative caregiver and care recipient outcomes have been repeatedly documented (Ding et al., 2022; Ge & Mordiffi, 2017; Schulz et al., 2020; Wolff et al., 2016). Although there are many reasons for family caregiver burnout, minimal help is one of the most significant (Gérain & Zech, 2019; Gibbons et al., 2014; Schulz et al., 2020). As the need for family caregivers of older community-based persons will increase in the years ahead, a mixed-methods investigation was carried out to determine the support needs and support services that family caregivers require when caring for community-based older family members with health or disability limitations. This 2022–2023 study was carried out in the Canadian province of Alberta. In 2021, the Canadian census revealed 629,220 Albertans were aged 65+, with 72,375 (11.5%) of these people 85+ years of age (Statistics Canada, 2022).

## Research Materials and Methods

After research ethical approval was gained, advertisements across the province of Alberta in community newspapers and community group websites were placed over April and May of 2022. These asked family caregivers to volunteer for a 6-month study involving weekly telephone interviews.

Among the 350+ persons who emailed a response, 150 were chosen through an intake telephone interview by the Principal Investigator to represent the expected diversity of family caregivers: Adults aged 65+ or younger (18–64), rural or urban residence, not working or worked in paid employment or own business, female or male, caring for one or more older family members who did not live in a nursing home or another congregate care facility, was a spouse or had another familial relationship, and lived with their care recipient or lived apart. The care recipients needed to be older adults (aged 65+) who required periodic or daily help, was cognitively competent or not, and was stable in health or not.

For informed consent, a letter of information about the study was sent electronically before each intake interview. This letter was discussed, and all questions answered. Each consenting caregiver was then asked to provide detailed information about themselves and the person(s) they cared for; information recorded in a secure database held by the Principal Investigator.

A verbal consent was also obtained before each weekly interview carried out by a research assistant. These interviews involved five standard questions:

(1) How are you?
(2) What did you do in the last week for the person/persons you help?
(3) Did you get any help this past week, and if so, was it helpful?
(4) Did you need any help this past week and not get it, and if so, what help did you need?
(5) Is there anything else you would like to mention or report?

All interviewers were nursing students. All interviews were conducted on a day and at a time negotiated between the research assistant and family caregiver. Every interview was reported on by the nursing students in a written format, with answers to the five questions highlighted. Many contained additional information, typically care recipient health and care need developments.

Written interview notes were emailed every weekend to the Principal Investigator, who printed and reviewed them. Each interviewed caregiver was then mailed or emailed a $20 gift card. This gift was intended to thank them for taking time each week for the interview. Interview reports often reflected their appreciation of this gift; it enabled them to take a few minutes to relax in a coffee shop or helped them buy food and essential care supplies. As no caregiver dropped out of the study, this gift likely helped retain family caregiver participants, a timeframe that could include bereavement in the event of a care recipient’s death. The relationships that developed between each caregiver and student nurse was another major factor. Most family caregivers were interviewed by the same research assistant over the six months.

The Excel spreadsheet database noted relevant interview information, such as identified caregiver needs, care recipient health changes, hospitalizations, and deaths or entry into a nursing home. The interview reports were also analyzed through a constant comparative approach to identify caregiver needs and essential support services. Over the first two months, 11 caregiver needs were identified, 10 before the care recipient's death and 1 post-death. The research assistants were asked to validate this list in the next 1–2 caregiver interviews. In the third month, afternoon and evening Zoom meetings were held with the research assistants and the family caregivers to seek their viewpoints on the identified 11 caregiver needs. No caregiver or student thought this list had unnecessary support needs listed or missed important needs.

### Literature Reviews

Following this validation, a PhD student was hired to conduct multi-database (Medline, CINAHL, PsycInfo, Directory of Open Access Journals, and JSTOR) research literature reviews on each of the 11 needs. English-language publications of all types of research investigations were sought (Munn et al., 2018). An annotated bibliography report of findings for each need was developed by the student and presented to the Principal Investigator, with full articles appended. After checking that no recent publications were missed, to ensure current as well as any long-established evidence, the findings were reviewed by the research team to determine if each need had been identified before. All had been researched and identified as important, often repeatedly. As such, the validity of the 11 caregiver support needs was established.

### Support Services Identification and Program Logic Investigations

The focus of interest then turned to the support services used by the family caregivers and appreciated, used but issues were present, or thought to be needed but were unavailable to them. All weekly interview reports were checked to create a draft list of support services, five in total. The research assistants were then asked to clarify, in the next 1–2 interviews, what support services the family caregivers used and if there were any issues with those services, and/or if they needed a support service and it was not available to them. All five support services were thus identified as commonly needed and or desired: Adult day programs (1 or 2 scheduled days each week where the care recipient left home to attend this program), respite services (a complete break from caregiving for a few hours or days), transportation (community bus/van with wheelchair and stretcher capacity), routine housekeeping and home maintenance (floor wash/vacuum, laundry, bathroom/kitchen clean, etc.), and personal care services provided in the home (bathing, dressing, medication management, foot care, ostomy care, denture/teeth cleaning, etc). In the sixth month, afternoon and evening Zoom meetings were held with the research assistants and caregivers about the list of five essential support services. No caregiver or student thought unnecessary services were listed or important support services were missed.

### Five Program Logic Investigations

Five program logic investigations were then conducted, one on each of the identified support services. “Program logic is a useful tool for engaging stakeholders in program planning” (New South Wales Government, 2017, p. 5). This work was carried out by the Principal Investigator, with the help of the same PhD student. In keeping with the aim of program logic investigations to learn how an organization or program/service works (Lawton et al., 2014), these investigations were done to determine who was providing these services, how and when each had been initiated, and how each operated. These investigations were also carried out to assess the potential for expansion.

In order to carry out the five program logic investigations, the PhD student obtained contact information for businesses, organizations, or individuals who were providing one or more of the services. Some advertised their services and some were identified through snowball techniques or caregiver interviews. Organizational representatives were then emailed and/or called to ask for an interview about their programs/services. One reminder occurred if there was no response in seven days. All subsequent interviews followed a discussed emailed information letter about the study and a verbal consent to participate. All interviews were conducted by the Principal Investigator, using a standard set of questions (see Appendix). These questions were developed and pilot tested in advance of the interviews. Each support service was investigated one at a time, with at least three respondents interviewed through telephone calls lasting 20–50 minutes. The findings were consolidated by the Principal Investigator into a brief report for review by the research team, with no corrections or concerns raised.

## Results

### Family Caregivers and Care Recipients

Despite efforts to include male caregivers, study participants were primarily female (133/150, 88.7%). Each cared for one or more family members who did not live in a congregate care facility. Two caregivers reported having a helpful spouse who willingly and regularly shared their caregiving duties. Half (*n* = 76, 50.7%) indicated occasional help from another family member, but only if asked for assistance. An almost equal number (*n* = 72, 48.0%) said no family members helped them.

The age of the family caregivers varied, from 22 (a female caring for her aunt while living with her and going to college) to 86 (a male caring for his 81-year-old wife). Nearly two-thirds were <65 (99/150, 64%), the official age of retirement in Canada, leaving one-third (34%, 51/150) 65+. Their average age was 66. A slight majority were working for income (78/150, 52%), with over half working full time (48/78, 61.5%). Twenty six indicated that they had gone to part-time employment so they would have time to be a caregiver. Four said they had always worked part-time, so that did not change when they became a caregiver. Notably, 56 of the 72 (77.8%) caregivers who were not working said they had worked until retirement, with retirement taken early so they could become a caregiver or so they could meet the increasing needs of their care recipient(s). One said, “I had always worked full time but started to realize how much help Mom was needing, and I began to think I could burn out and get ill myself. I made the decision to quit work as I could financially manage that and I could not let my Mom down. She needed me.”

The 150 caregivers cared for 177 people; 23 (15.3%) cared for more than one family member. Four helped 3 people (mother and father, and mother-in-law or father-in-law) and 19 helped two people. Sixteen of these 19 cared for a female and a male, in all cases a mother and father or step-father. The remaining 3 each cared for 2 females. Two cared for their mother and her sister, and one helped her mother and her mother’s long-term friend who had moved in to help her mother and then began to need help herself, a person described as an “adopted Aunt.”

Slightly more than half lived in a separate dwelling from their care recipient(s) (82/150, 54.7%); the remaining 45.3% (68/150) lived together with their care recipient(s) in the same house or apartment/condominium. Five reported they built a multi-generational home or renovated one so they could live together. Whenever caregivers and their care recipient(s) did not live together, all but 6 of the 82 lived close to each other. Most reported that they only needed a short walk to reach the care recipient’s home or a short (5–30 minutes) drive to reach the care recipient’s home. In three cases, the care recipient sold a farm or house and moved into an apartment, with proximity to their family caregiver a key feature in this move.

Six of the 150 caregivers (4%) reported having a 1–3 hour drive to reach the home of their care recipient; all parents. Three travelled on weekends to stay at the care recipient’s home for 1–4 days. In two cases, this was the original farm home and in one case it was the original family home in a town. These three caregivers also made occasional trips during the week when necessary and frequent mid-week calls. The remaining three caregivers reported a 1 hour drive to reach their care recipient’s home. They made 2–3 trips per week, with visits lasting 2–4 hours.

Of all 177 care recipients, 71 (40.1%) were male; most were female (106/177, 59.9%). Their ages ranged from 65 to 101 (mean = 83.9). The help required varied, from needing to be driven somewhere or needing help with other instrumental activities of daily living such as maintaining the home and yard, to complete 24/7 care. No differences in care requirements based on care recipient age were noted. It was commonly reported however that a progressive increase in care needs was noticed over time. The recorded weekly information also demonstrated increasing care needs. Most often, this caregiving starting with needing help with physically demanding and then with less physically demanding instrumental activities of daily living (i.e., driving, banking, home maintenance including routine or periodic cleaning such as vacuuming and yard work, and basic home repair activities such as painting walls and replacing light bulbs). This typically progressed to including basic activities of daily living (i.e., getting out of bed and getting back into bed, dressing, toileting, and bathing) and then to less strenuous activities of daily living (i.e., eating and taking medications). In five cases, a sudden major decline in health had occurred, with the care recipient returning home from a hospital with much greater care needs. These included the use of technologies such as a urinary catheter or ostomy.

Six of the 177 (3.4%) care recipients were admitted to a nursing home over the 6-month study. All six caregivers reported a major change in their life activities because of this move. They recognized that they were having to adapt to a new life. Four had provided care recipient support for more than two years and two had provided support for over one year. In all six cases, this move occurred because of 24/7 care needs, including personal care. Four care recipients had cognitive impairment, with two wandering such that they were not safe outside of a secure facility.

Twelve of the 177 care recipients (6.8%) died. All of their caregivers continued to be interviewed, but the weekly interview questions were shifted to focus on them. All 12 appreciated being asked how they were doing, and if they had gotten any help following the death or if they had needed help and did not get it. These interviews revealed that they were grieving not only the death of a loved one but also the loss of a social role, as they had taken on the identity of a family caregiver. One said, “It was like I lived to be Mom’s caregiver, that kept me busy every day, and then she was gone, and I am lost; everyone knew me as Mom’s caregiver and I’m not that anymore.” Another reported that she had taken a 3-month leave from work when her mother became terminally ill, and she was expected to report back to work immediately after the funeral. She said was a “huge” adjustment for her as she was actively grieving and adjusting to a life that was no longer scheduled around her mother’s care needs. She said, “It is like shifting gears from one life to another, at a time when I am so tired from caregiving and now so grieving her and our life together.” Another caregiver reported, “I was so used to having no spare time looking after Dad, and now suddenly, it is like I have nothing to do. Filling in time is hard. I have not talked to friends for a long time. I retired because Dad needed me. I lost contact with a lot of people.”

### Identified Caregiver Support Needs

As indicated above, 11 family caregiver support needs were identified: Information, emotional support, help with care recipient instrumental activities of daily living, help with care recipient activities of daily living, respite, self-care encouragement, transportation assistance, financial help with care-at-home costs, anticipatory grief of the family caregiver, anticipatory grief of the care recipient, and support after their caregiving ends. Each was examined for existing evidence through a scoping research literature review. Each had been studied before, often repeatedly. For instance, information needs were commonly found among caregivers (Hall & Holtslander, 2022; Prevo et al., 2018). Past research often found caregiver emotional support needs existed (Arriagada, 2020; Mento et al., 2019; Ong et al., 2018; Teixeira et al., 2020). Multiple research investigations found caregivers needed help with their care recipient’s instrumental activities of daily living (Beach & Schulz, 2017; Tamura et al., 2023). Needing help when care recipients had basic activities of daily living limitations was also identified in multiple research reports (Lin et al., 2019; Schmidle et al., 2022). Caregiver respite needs were similarly identified in numerous research reports (Aung et al., 2021; Prevo et al., 2018; Roberts & Struckmeyer, 2018). Needing encouragement to take care of one’s own self was identified in research focused on caregiving (Freedman & Spillman, 2014). Needing help with care recipient transportation was identified as an important community-based care issue (Ravensbergen, 2020; Riffin et al., 2017; Tamura et al., 2023). Needing financial help for care-at-home costs was identified in multiple studies (Darabos & Faust, 2023; Mah et al., 2021; Marani & Allin, 2022). Anticipatory grief of the family caregiver, as a consequence of knowing that the death of the care recipient was approaching, was identified in palliative research investigations (Coelho et al., 2018; Li et al., 2023; Treml et al., 2021). Anticipatory grief of the care recipient, illustrated by their refusing help and refusing to move from a home that was no longer safe for them, had been identified before (Bilić et al., 2022). Needing support after the caregiving ends was also commonly found (Arriagada, 2020; Chan et al., 2023; Lohrasbi et al., 2023).

### Essential Support Services

As indicated above, five caregiver support services were identified: Adult day programs, respite services, transportation, routine housekeeping and home maintenance, and personal care services provided to the care recipient in the home. The adult day programs were described by those caregivers who had these available as full days scheduled for 1 or 2 set days each week. Most often, a community van or the day program organization’s van transported the care recipient. These days were reported as important opportunities for the care recipient to get away home and socialize with others, and take part in mentally stimulating activities such as singing or playing cards. Additional services were occasionally available, such as foot care, banking, legal services, and primary healthcare assessments or healthcare follow-ups. The caregivers also reported a much-needed daylong break to work for income, clean homes, shop, or carry out activities that were difficult to do when the care recipient was at home or was accompanying them.

Respite services lasting a night or a few days were also identified as essential, but only five family caregivers reported used a respite service. Three had obtained publicly funded respite home care services for 24 hours so that they could be away overnight. The remaining two had a local nursing home or rural hospital available for 1–7 day booked respite breaks. Both caregivers appreciated having this respite to go on a needed holiday. They said that they did not worry about their family member as they believed they would get good care and be kept safe. Many other caregivers instead said that they had weddings or funerals they could not attend, as they could not take their care recipient(s) with them and they could not be left at home alone.

Transportation was another major needed service. Most caregivers reported how difficult it was to get their weak or disabled family member into a car or truck and take them somewhere in town or out of town; often for a doctor, dentist, or legal/banking appointment. Care recipients typically had difficulty walking and in some cases were confused and could not be talked into getting ready and/or getting into a vehicle. Some trips were painful for the care recipient and the family caregivers were distressed by this. Most trips away from the care recipient’s home were short, but some were 3–6 hours long, often to see a medical specialist in a distant city. Only eight caregivers reported having a community van with wheelchair or stretcher capacity available for these journeys. Difficulty was often experienced in using these however, as they could not be booked or rebooked on short notice.

Routine housekeeping and basic home maintenance comprised another essential support service. Most caregivers reported having little time for kitchen and bathroom cleaning, vacuuming, laundry, and minor home repairs, as their priority was their care recipient’s direct care needs. Moreover, nearly half had two homes to clean and maintain. When the caregiver worked outside of the home, this meant having fewer hours to carry out these activities. Moreover, some were disabled. One 80-year-old caregiver reported: “I care for my 85-year-old husband who can hardly walk and I can hardly walk. I need knee and hip surgery; it is painful for me to get even the most basic things done around our home.”

Personal care services needed by the care recipient in their home was the fifth identified essential service. This service was identified as the most essential by some caregivers. One 84-year-old woman said she could not help her husband into the shower as he was so weak. Because he was often incontinent, he needed a shower every day, if not multiple times each day. Most of the 177 care recipients were said to require help with bathing or showering, getting in and out of bed, dressing and undressing, and walking around the home such as to and from the toilet. The caregivers were not always available for these activities, as was the case for those who did not live with their care recipient(s). Home care support for the care recipient’s personal care needs was seen as essential by these caregivers. Only 3 of the 150 caregivers indicated that they had enough publicly funded home care to address the care needs of their family member. Nearly half of the remaining 147 caregivers (*n* = 69, 46.9%) reported having some publicly funded homecare support, but all indicated that it was very limited in terms of hours each week and in what the homecare worker could do. One said, “the homecare worker is allotted 30 minutes every morning to care for Mom, five days a week, that includes getting her out of bed and into the tub and getting her dressed. I don’t know how she can do that, as Mom is so slow moving. That homecare worker cannot clean the bed, even if Mom had soiled it, or clean the tub.” Some caregivers also reported no homecare support was provided over the night or on weekends, this meant they often lost sleep and were tired.

Although the 177 care recipients could have been moved into a senior’s care facility, most caregivers said that they did not want them to go into a nursing home. Care recipients were also said to want to stay at home. However, 10 of the 150 caregivers applied for a nursing home entry to take place in the future if necessary. Most caregivers said they hoped they would receive some or more publicly funded home care if care needs increased.

Only 6 of the 177 care recipients moved into a nursing home. Among these was the only one said to be wealthy; his wife reported he could easily afford to pay privately for 24/7 home care. He first had a full-time caregiver who came to the home every day of the week for 8-hour shifts. When they could not find a nighttime caregiver after he began to need 24/7 care, he was moved into a nursing home as an application had been made previously on the advice of a home care nurse. They did not feel they could turn this admission down when the bed became available, as it was for a local nursing home and so was easily accessible for daily visits by the wife. He died shortly after this move, and his wife reported she was glad to have “keep him home as long as possible.”

#### Program Logic Findings

As indicated above, each of the five identified supports was explored through a program logic investigation. All supports were found to be provided by small independent businesses operating in select regions of the province. Some were charities or non-profit organizations; the majority were small for-profit businesses. In all cases, they relied on government grants that they had applied for and had successfully obtained. These grants funded specific defined services. Regular reporting was expected on their achievement of contract terms. All grants were limited to 1 or 2 years of funding support, and then a reapplication was needed. This reapplication was said to be time consuming and expensive as most hired a grant writer to do them. Three respondents reported being unsuccessful in getting a replacement grant. They had to call clients to tell them that their service would no longer be provided. One recalled telling clients that their publicly subsidized home cleaning service would end next week. The option now would be for them to find, supervise, and pay cleaners privately, or go without.

## Discussion

This study focused on what family caregivers of community-dwelling frail-elderly and other older family members with dependency care needs require. Family caregivers are often considered the most important component of consistent and essential community-based support for older adults who cannot independently perform activities of daily living (Schmidle et al., 2022; Tamura et al., 2023). Yet, this caregiving is often unsupported. Eventually, the care recipients near the end of life, a time of great risk for being institutionalized in a nursing home or hospital (Bornet et al., 2021). The work of family caregivers of older community-dwelling family members, who are primarily lay people, is thus done despite the impending death of their loved one (Dehpour & Koffman, 2023; Treml et al., 2021). The 11 support needs identified through this study include not only what family caregivers commonly could use when helping older family members but also what they need following their death.

This study is only the latest to highlight the work of family caregivers. A great deal of research information already exists on family caregivers and caregiving, including (a) who family caregivers are, typically a middle-aged to older spouse (Arriagada, 2020; Hazzan et al., 2022), (b) how much time they devote to caregiving, which often starts with a few hours each week and then often progresses to 24/7 care (Schulz et al., 2020), (c) what skills and knowledge family caregivers need to gain in order to care for frail-elderly and other older dependent people, such as in providing hands-on care with bathing, home maintenance and shopping, banking and financial management, transportation, health and medication monitoring, communication duties, and advocacy (Gibbons et al., 2014; Ravensbergen, 2020; Tamura et al., 2023), and (d) caregiver burnout and illness rates, with many caregivers become overburdened, if not ill, over the amount of care that they often alone provide and often over many months or years (Gibbons et al., 2014; Schulz et al., 2020). The question then is what help should be provided to family caregivers so more can begin and more can continue to carry out caregiving duties until death occurs.

One notable finding was that many caregivers have more than one family member needing help. Arguably, these care needs must be met to prevent nursing home entry and prevent hospitalizations for avoidable injuries and unaddressed health concerns. Expecting family caregivers to provide community-based care alone, or with few publicly funded supports, is no longer a viable option in countries where advanced aging is common. Unfortunately, the 150 interviewed family caregivers living in rural and urban areas of Alberta often reported little to no help. Assured access to the five identified supports would not only benefit the care recipient but also the caregiver as these could prevent caregiver health issues, maintain family relationships, and enable good deaths. Good deaths would help caregivers avoid suffering overly severe, prolonged, and impactful grief (Wilson et al., 2019).

Despite the benefits of community-based care services, finding sufficient financial support for them is a concern in Canada as most health system spending is oriented to acute care services provided in medical clinics and hospitals (Allin, 2022; Alberta Health Services, 2022). It is often assumed that older people are high users of hospitals, yet <14% are hospitalized each year (Wilson et al., 2024). This percentage is important; the 2021 Canadian census revealed 7.0 million (19%) Canadians were aged 65+ and 861,000 (11.8%) were aged 85+ (Statistics Canada, 2022). Moreover, only 3–4% of older Canadians move into a congregate-care facility providing 24/7 care (Statistics Canada, 2017). Unquestionably, more Canadians are reaching an age where they will be “frail” and vulnerable to health issues such as infections, falls, cancer, and worsening chronic illnesses (Dent et al., 2019; Fong, 2019). These people often begin to need help with activities of daily living (Arriagada, 2020; Schmidle et al., 2022; Tamura et al., 2023). Proactive community-based care should become a priority (Khandelwal et al., 2012).

One of every four adult Canadians is providing dependent family member care (Statistics Canada, 2022). This caregiving has minimal financial or practical support (Duncan et al., 2016; Marani & Peckham, 2023). Our study determined 11 needs of caregivers and 5 essential services that should be available regardless of where they and their care recipients live and also regardless of any personal financial capacity to pay privately for community-based support services.

Only 1 of our 150 family caregivers was clearly able to pay for essential support. An earlier study of 1.2 million midlife and later-life family caregivers in Canada revealed they spent a total of 12.6 million out-of-pocket dollars when caring for loved ones with chronic illnesses and complex care needs (Duncan et al., 2016). About half of Canada’s caregivers occupy this role for at least four years and one-third do so while still working (Sinha et al., 2018). Scarce public funding for community-based care is the most important reason for this out-of-pocket spending (Marani & Peckham, 2023). Gender issues are a related consideration. Not only do females typically live longer than males so they are more at risk of end-of-life disability, but family caregivers are most often female. Our study’s recruitment of mostly female caregivers illustrates this.

All Canadians can be thankful that medically necessary physician and nurse practitioner care, as well as hospital-based care services, are publicly funded (Arriagada, 2020). Hospitals are very expensive, with hospital admission prevention through proactive home care services and hospital-at-home services needing to become a major focus of action. In Canada, “care closer to home” options tailored towards the needs of caregivers and care recipients were envisioned in the 2015 *National Seniors Strategy* developed by the National Institute on Ageing (2020). In the continuing absence of a federal community-care strategy (Sinha et al., 2016), dollars invested in home and community-based services persistently pale in comparison to those earmarked for institutional care (National Institute on Ageing, 2020). Notably, one of the three top sources of financial strain for family caregivers is transportation (Marani & Peckham, 2023; Seidman, 2023). Our study found transportation support was one of five family caregiver needs. Not only was transportation a financial concern, but it was also a care concern given that transportation in a personal car or truck was often uncomfortable. Some trips were not likely made, even if essential.

One of the key findings of the program logic investigations was that each service is not universally available across Alberta. Alberta is not alone; no province appears to have a consistent set of home-based and community-based support services available to all persons with dependency care needs. Until the five identified support services are universally available, family caregivers and care recipients will be at risk of adverse events and outcomes. Currently, home care programs, providing a limited range and amount of personal care services, comprise the only universal community-based program across Canadian provinces (Arriagada, 2020; Statistics Canada, 2022).

With all provinces struggling to pay for medically necessary healthcare services as these typically comprise one-third of total government expenditure (Statistics Canada, 2020), the federal government should develop a policy for an annual federal-to-provincial funding transfer so each province can provide a set of five community-based support services for all family caregivers and care recipients. This policy is not a new concept. Since 1966, the USA’s Older Americans Act enacted federal-to-state funding transfers designed to fund community-based services for enabling older people to age in place (Centers for Medicare & Medicaid, 2023). Moreover, the 1985 Hospice Medicare Act was passed to support dying Americans at home and prevent hospitalizations (Centers for Medicare & Medicaid, 2023). These two policies provide a blueprint for Canadian policy developments.

Assured aging-in-place support services across Canada will require earmarked funding (Marani & Peckham, 2023). Moreover, community-based care should be designed using an integrated service approach, where the aim is to provide the right care, in the right place, and at the right time (Organization for Economic Co-operation and Development, 2023). Integrated care planning will require caregiver input (Barrenho et al., 2022; Canadian Home Care Association, 2019). Exemplars such as the around-the-clock Virtual Long Term Care Home program for Ontarians and Saskatoon’s Home First program (Compton et al., 2020) exist.

### Limitations

This study was carried out in one Canadian province; it does not represent what may be the case elsewhere. Another limitation is that data were primarily gained from female caregivers. Few male caregivers volunteered for this study despite repeated advertisements in the hope that more would hear of this study and volunteer. It is likely though that this reflects gender-based family caregiving and life expectancy differences.

## Conclusions

Given population aging, it is time to recognize the essential role of family caregivers in supporting older community-based family members who need help to avoid nursing homes and hospitals. This study identified 11 needs that family caregivers commonly encounter when providing community-based care for one or more older family members. Five essential supports were also identified. A patchwork of intermittent support services was found to exist in the province of Alberta, a situation that is likely elsewhere. Two policy needs were identified: (a) provincial policy assuring family caregiver and care recipient access to the five supports and (b) federal policy ensuring a federal-to-provincial funding transfer will meet the cost of these provincially provided services.

## Supplemental Material

Supplemental Material - Identifying Needs and Support Services for Family Caregivers of Older Community-Based Family Members: Mixed-Method Research Findings Supplemental Material for Identifying Needs and Support Services for Family Caregivers of Older Community-Based Family Members: Mixed-Method Research Findings by Donna M. Wilson, Jennifer Heron, and Gilbert Banamwana in Journal of Applied Gerontology
