# Supplementary material for: Identifying Needs and Support Services for Family Caregivers of Older Community-Based Family Members: Mixed-Method Research Findings
Source: J Appl Gerontol. 2025 Jan 9;44(9):1424–34. doi: 10.1177/07334648241308726 (PMC12335626; doi:10.1177/07334648241308726)
Supplement: Supplemental Material - Identifying Needs and Support Services for Family Caregivers of Older Community-Based Family Members: Mixed-Method Research Findings [file sj-pdf-1-jag-10.1177_07334648241308726.pdf]

# Appendix. Questions for Service Provider Organizations

1. What service or services do you provide?
2. How long have you been in business?
3. How and why did your business get started?
4. How are you funded?
5. Where are you located, and where are your services provided?
6. How many people work for you? And in what capacity?
7. Could you expand to have more clients/what would it take for you to increase the number of clients?
8. Could you expand and offer additional (new) services, and if so, what would it take for you to offer these additional services?
